# Postbiotics Formulation and Therapeutic Effect in Inflammation: A Systematic Review

**DOI:** 10.3390/nu17132187

**Published:** 2025-06-30

**Authors:** Kinga Zdybel, Angelika Śliwka, Magdalena Polak-Berecka, Paweł Polak, Adam Waśko

**Affiliations:** 1 Department of Biotechnology, Microbiology and Human Nutrition, Faculty of Food Science and Biotechnology, University of Life Sciences in Lublin, Skromna 8, 20-704 Lublin, Poland; angelika.sliwka@up.lublin.pl (A.Ś.); magdalena.polak-berecka@up.lublin.pl (M.P.-B.); adam.wasko@up.lublin.pl (A.W.); 2 The Provincial Specialist Hospital in Biala Podlaska, Terebelska 57-65, 21-500 Biala Podlaska, Poland; pawel.polak@szpitalbp.pl

**Keywords:** postbiotics, probiotics, non-live bacteria, inflammatory diseases, dysbiosis, gut microbiome, anti-inflammatory effects

## Abstract

**Background**: Postbiotics are bioactive compounds derived from inactivated probiotic microorganisms that show potential for preventing and treating inflammatory diseases. This review aimed to evaluate the evidence on their therapeutic effects in inflammatory conditions. **Methods**: A search of PubMed, Scopus, and Web of Science databases from 2014 to 2024 identified 39 eligible studies. Article selection was conducted using the Rayyan platform, risk of bias was assessed with the Cochrane ROB 2 tool, and results were visualized with ROBVIS. Bibliometric networks were constructed using VOSviewer. Due to data heterogeneity, a meta-analysis was not performed; therefore, results were described and presented graphically. **Results:** The most commonly used microorganisms belonged to the *Lactobacillaceae* and *Bifidobacteriaceae* families, with heat inactivation as the predominant method. Postbiotics exert multifaceted anti-inflammatory effects by modulating cytokine expression, influencing immune cell signaling pathways, and strengthening epithelial barrier integrity. They regulate immune mechanisms such as the Th1/Th2 and Treg/Th17 balance, indicating their potential in treating inflammatory bowel diseases, autoimmune diseases, and metabolic syndrome. However, the heterogeneity of studies, their limitations, and risk of bias require cautious interpretation. **Conclusions**: Future research should focus on standardizing postbiotic preparations, conducting long-term clinical trials, and analyzing synergistic effects of different strains. Postbiotics offer a promising approach to managing inflammation, with potential applications in functional foods and nutraceuticals.

## 1. Introduction

Postbiotics are a novel category of bioactive compounds derived from probiotic microorganisms that offer potential health benefits without the need for live microbes to be present. The International Scientific Association for Probiotics and Prebiotics (ISAPP) defines postbiotics as “preparations of inanimate microorganisms and/or their components that confer a health benefit on the host” [1]. This definition distinguishes postbiotics from probiotics and prebiotics, emphasising their microbial origin and non-viable nature. Postbiotics encompass various components, including microbial metabolites, cell wall fragments, short-chain fatty acids (SCFAs), extracellular polysaccharides, peptides, and other cellular components [2,3] These compounds can modulate immune responses, enhance gut barrier function, and alter microbial ecology [4,5,6].

The growing scientific interest in postbiotics has led to numerous preclinical and clinical studies investigating their potential therapeutic effects in various health contexts. Emerging evidence suggests that postbiotics may have beneficial effects on gastrointestinal diseases, metabolic diseases, atopic conditions, and even neuropsychiatric conditions by modulating the gut–brain axis [7,8,9,10]. One of the main mechanisms of action of postbiotics is their significant anti-inflammatory activity, which is attributed to the various components and bioactive fractions present in postbiotics [11]. A growing number of laboratory and clinical studies have suggested that chronic inflammation can lead to permanent damage to healthy organs, tissues, and cells, thereby increasing the risk of many common and fatal diseases. Because it plays a key role in the development and progression of various diseases, the development of effective prevention methods may provide promising support for anti-inflammatory therapies [12]. However, the current body of literature remains fragmented, with diverse formulations, target conditions, and outcome measures. This diversity limits the ability to draw clear conclusions regarding their efficacy and clinical relevance.

Despite the growing research in this field, there is a lack of comprehensive systematic reviews synthesising the current evidence on postbiotic formulations and their therapeutic outcomes in chronic inflammatory conditions. Both in vitro and in vivo studies indicate that postbiotics are a promising approach for the prevention of inflammatory diseases by exerting a range of bioactivities, including antioxidant and immunomodulatory activities, modulation of the gastrointestinal microbiota, and enhancement of epithelial barrier function. However, the basic signalling pathways involved in their action remain incompletely understood and require further research [13]. This gap highlights the need for a structured overview of the field, identification of knowledge gaps, and guidance for future research and clinical applications of postbiotics to prevent and support the treatment of inflammatory conditions [14,15].

The potential advantages of postbiotics over traditional probiotics include enhanced stability, standardisation, and safety. Unlike live probiotics, postbiotics are generally less affected by factors such as temperature, pH, or storage conditions. However, these factors can still influence the stability of certain biomolecules, such as LPS, depending on the specific range. Higher stability of postbiotics allows for easier incorporation into various food products and supplements, potentially expanding their applications in the food and pharmaceutical industries. Additionally, the use of non-viable microbial components may reduce the risk of adverse effects associated with the administration of live microorganisms, particularly in immunocompromised individuals or those with a compromised gut barrier function [16].

Research on the mechanisms of action of postbiotics has revealed multiple pathways through which these compounds exert beneficial effects. For instance, certain postbiotic components have been shown to interact with pattern recognition receptors on immune cells, modulate inflammatory responses, and enhance innate immunity. Short-chain fatty acids, a common class of postbiotics, have been demonstrated to influence gene expression, metabolism, and cell function through various mechanisms, including histone deacetylase inhibition and G-protein–coupled receptor activation. Furthermore, some postbiotics have been found to exhibit antimicrobial properties, potentially contributing to the maintenance of a balanced gut microbiota and protection against pathogenic microorganisms [17].

The diversity of postbiotic formulations and their potential applications presents both opportunities and challenges for researchers and clinicians. Although the range of bioactive compounds allows for targeted interventions in various health conditions, it also necessitates careful characterisation and standardisation of postbiotic preparations. Future research should focus on elucidating the specific bioactive components responsible for the observed health benefits, optimising dosing regimens, and investigating the potential synergistic effects of different postbiotic compounds. In the context of inflammation, research on the effects of postbiotics on the modulation of the inflammatory response is of particular importance. Elucidating the specific mechanisms of their action on the immune response at the cellular and molecular levels may contribute to the development of more effective and safer treatments for inflammatory diseases [18].

Therefore, this systematic review was conducted to evaluate and synthesise the current evidence on postbiotic formulations and their therapeutic effects in human and animal models of inflammation. The objective was to identify common patterns in formulation strategies, classify reported clinical or physiological outcomes, and critically assess the evidence quality. Through this approach, we seek to provide a structured overview of the field, highlight existing knowledge gaps, and inform future research directions and clinical applications of postbiotics in the context of supporting anti-inflammatory therapies in the body.

## 2. Materials and Methods

### 2.1. Protocol

The systematic review protocol was established based on the Preferred Reporting Items for Systematic Reviews and Meta-Analysis Protocols (PRISMA) statement [19]. To identify key research areas on probiotics, postbiotics, and gut microbiota, keyword co-occurrence analysis was conducted using VOSviewer (version 1.6.20) tool [20].

### 2.2. Eligibility Criteria

To be eligible for inclusion, the studies had to include information on the use of postbiotics (formulation and supplementation) in human and animal models. These studies also had to contain information on the use of inactivated microorganisms for the treatment of different inflammation-related diseases in the body and their mechanisms of action. Studies on live microorganisms, in vitro research, reviews, and those published only as abstracts were excluded.

### 2.3. Sources of Information and Search Strategy

Systematic literature searches were performed using the following electronic bibliography databases: PubMed, Scopus, and Web of Science, from 2014 to December 2024, without language restrictions on English. The literature search was based on keywords defined by the authors of this study. The selected search algorithm was as follows: ((((((Postbiotics)) OR (Postbiotic formulations)) OR (Postbiotic supplementation)) AND (((bacteria treatment) OR (microorganisms)) OR (formulation))) AND ((dysfunction) OR (diseases))) AND (((((therapeutic effects) OR (clinical trials)) OR (health)) OR (treatment)) OR (therapy)) NOT (review). The search algorithm was tailored according to the structure of each database. One researcher downloaded the Research Information Systems (RIS) file generated by each database and uploaded it to the VOSviewer software tool, which enabled the construction and visualisation of bibliometric networks. The Research Information Systems (RIS) file generated by each database was also uploaded to the Rayyan^®^ web application for systematic reviews [21].

### 2.4. Selection Process

The selection of articles for inclusion in the review was conducted in two stages using Rayyan^®^, which allows reviewers to blind the selection process. In the first stage, titles and abstracts were analysed based on the inclusion criteria. In the second stage, full-text articles were assessed to confirm their eligibility. At both stages, each article was independently reviewed by three researchers. In cases of disagreement, the study was reassessed and resolved through discussion.

### 2.5. Data Extraction

Three authors independently extracted data from each included study using the Rayyan^®^ platform. The extracted data included the first author, year of publication, and methodological details, such as the type of study conducted, population (species, age, sex, and sample size), type of disease studied, intervention (species or strain of microorganisms, method of inactivation, postbiotic formulation, dosage, and duration of administration), and outcomes (method of application and mechanisms of action of postbiotics in different diseases).

### 2.6. Risk of Bias in Individual Studies

The risk assessment for each study was conducted independently by three authors using the Cochrane Risk of Bias Tool (ROB 2) [22]. The ROBVIS tool was also used to visualise risk assessments [23]. As required by ROB 2, the following parameters were independently assessed: (1) risk due to the randomisation process; (2) risk due to deviations from the intended interventions; (3) risk due to missing outcome data; (4) risk due to the measurement of the outcome; and (5) risk due to the selection of the reported result. Tool ROB 2 was used to analyse the overall risk of bias, represented as high risk in red, risk uncertainty in yellow, and low risk in green. Any differences in bias assessment were resolved through discussions among the three authors until an agreement was reached.

### 2.7. Data Synthesis

Meta-analysis was not possible due to the heterogeneity of the included studies, which was due to several factors. First, the review included both human and animal studies. Second, the studies included both single-strain and multi-strain postbiotic preparations. Third, the formulation of postbiotics and the forms of administration were diverse. Furthermore, the methods used to assess the therapeutic potential of postbiotics varied between studies. The duration of the intervention also varied greatly, from a few days to a year. Therefore, the results were presented in the form of structured graphs divided into groups (type of study, disease, single-strain postbiotic preparations, multi-strain postbiotic preparations, inactivation method, formulation, and method of administration). All data within groups were summed and presented as percentages.

## 3. Results

### 3.1. Summary of Studies

**Figure 1:**
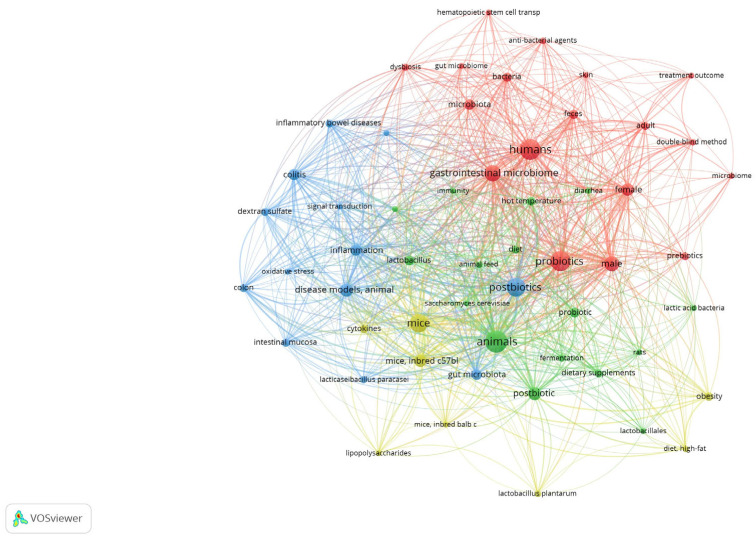
Co-occurrence networks of all 38 keywords that appeared at least 7 times. The keyword networks are coloured according to the four clusters generated, which indicate inter-relationships.

To ascertain the predominant research domains in the literature concerning probiotics, postbiotics, and gut microbiota, a co-occurrence analysis of keywords was performed using VOSviewer software. This analysis encompassed the terms present in the titles and abstracts of the publications selected for review. The findings are shown in Figure 1. The resulting map revealed four distinct thematic clusters, illustrating the interdisciplinary nature of the study area. The red cluster is centred on clinical research involving humans. Dominant terms, such as humans, gastrointestinal microbiome, microbiota, probiotics, females, and males, indicate a broad interest in the impact of gut microbes on human health, including sex differences and intervention designs (e.g., the double-blind method). The blue cluster encompasses inflammatory and immune-related topics of research. The presence of terms such as inflammatory bowel disease, colitis, oxidative stress, and inflammation confirms the significance of preclinical research in analysing the mechanisms of action of probiotics and postbiotics in the context of gastrointestinal pathology. The green cluster represents the domain of animal experiments. The frequent co-occurrence of terms such as animals, mice, gut microbiota, postbiotics, and dietary supplements indicates the use of animal models to assess the function and efficacy of microbiological interventions. The yellow cluster focused on the biotechnological aspects of formulations and active ingredients. Terms such as *Lactobacillus plantarum*, cytokines, lipopolysaccharides, and obesity reflect the interest in specific bacterial strains, their metabolites, and their effects on metabolic and immunological parameters of obesity. Many studies on probiotics, postbiotics, inflammation, and the gastrointestinal microbiome have confirmed the centrality of these issues in the current scientific discourse. The density of connections and overlapping clusters suggests an intense integration of clinical, experimental, and technological perspectives.

**Figure 2:**
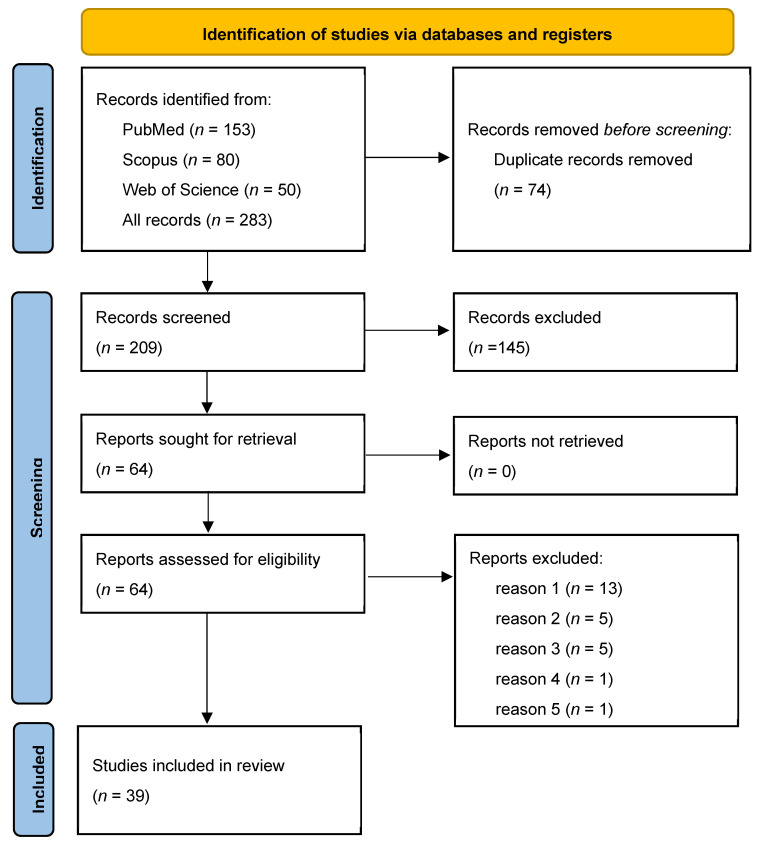
PRISMA flow diagram summarising the article selection process and reasons for exclusion: reason 1—lack of information on inactivation method (*n* = 13); reason 2—lack of information on the microorganisms used (*n* = 5); reason 3—not available full text of publication; reason 4—other medical diseases (*n* = 1); reason 5—studies that are in vitro experiments (*n* = 1). PRISMA, Preferred Reporting Items for Systematic Reviews and Meta-Analysis.

Publication selection for this systematic review was conducted in accordance with the PRISMA guidelines. A total of 283 bibliographic records were identified in 3 databases: PubMed (*n* = 153), Scopus (*n* = 80), and Web of Science (*n* = 50). After the removal of 74 duplicate records, 209 unique items were screened. An analysis of the titles and abstracts led to the exclusion of 145 publications, resulting in 64 full-text articles being evaluated for eligibility. Of these, 25 studies were excluded from further analysis for the following reasons: lack of information on the inactivation method used (*n* = 13), lack of information on the microorganisms used (*n* = 5), unavailable full text of publication (*n* = 5), other medical diseases (*n* = 1), and studies that were in vitro experiments (*n* = 1). Ultimately, 39 studies met the eligibility criteria and were included in the review. The details of the selection process are shown in the PRISMA diagram (Figure 2).

The following summary presents the data extracted from Table 1, which delineates the characteristics of the studies included in this systematic review, with a particular focus on the type of microbiota-based interventions, administration routes, microbial strains used, and clinical contexts.

**Figure 3:**
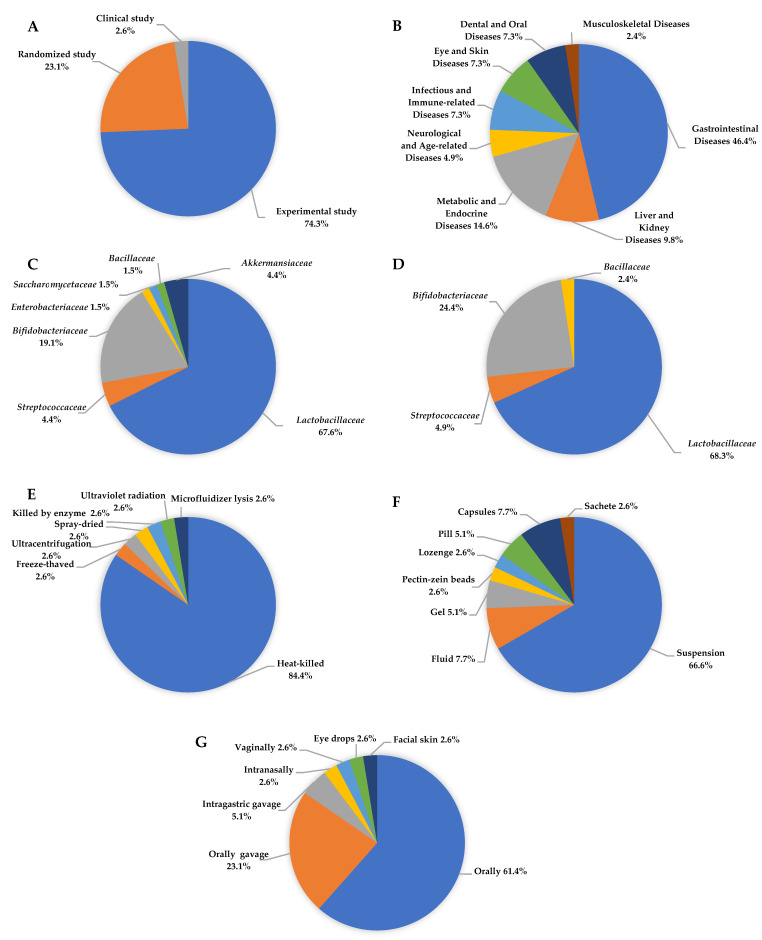
Characteristics of postbiotics used in research on inflammatory disease: (**A**) Study design, (**B**) type of disease, (**C**) single-strain postbiotics, (**D**) multiple-strain postbiotics, (**E**) method of inactivation, (**F**) forms of postbiotics formulation, (**G**) postbiotics applications method.

Among the 39 studies analysed, experimental design was predominant (74.4%), with a significant proportion of randomised trials (23.1%), indicating a relatively high methodological standard in the field of microbiota-targeted interventions (Figure 3A). The interventions addressed a broad spectrum of clinical conditions associated with inflammatory responses (*N* = 41), most frequently gastrointestinal diseases (46.3%), followed by metabolic and endocrine diseases (14.6%), liver and kidney diseases (9.8%), and infectious, dermatological, oral, neurological, and musculoskeletal conditions (Figure 3B). In the subset of single-strain interventions (*N* = 68), the most frequently used genera included *Lacticaseibacillus*, *Lactiplantibacillus*, and *Limosilactobacillus*, belonging to the family *Lactobacillaceae* (67.6%). The second and third most frequently used bacteria were from the *Bifidobacteriaceae* (19.6%) and *Akkermansiaceae* (4.4%) families, respectively. The most common individual strains were *L*. *plantarum*, *L*. *casei*, *B*. *animalis*, and *S*. *thermophilus* (Figure 3C). Multi-strain formulations typically contained three to eight strains, most frequently combining *L*. *casei*, *L*. *plantarum*, *B*. *animalis*, *B*. *longum*, and *L*. *reuteri*, reflecting a preference for well-characterized strains from the *Lactobacillaceae* and *Bifidobacteriaceae* families due to their established safety profiles and immunomodulatory potential (Figure 3D). Regarding inactivation methods, heat-killing was the most frequently employed technique (84.6%), including sterilisation, pasteurisation, and tyndallization, whereas other methods such as freeze-thawing, ultracentrifugation, spray-drying, enzyme treatment, and UV radiation were rarely used (2.6% each) (Figure 3E). Suspensions were the most commonly used delivery form (66.7%), followed by fluids and capsules (7.7% each), with pills, gels, lozenges, and advanced carriers, such as pectin-zein beads, appearing in isolated cases (Figure 3F). Oral administration was the predominant route of administration (61.5%), with additional studies employing oral (23.1%) or intragastric gavage (5.1%). Alternative administration routes, such as intranasal, vaginal, ocular, or topical, were reported infrequently (2.6% each), indicating the primary intestinal target of postbiotic interventions, while also highlighting the emerging interest in systemic or localised extraintestinal effects (Figure 3G).

**Figure 4:**
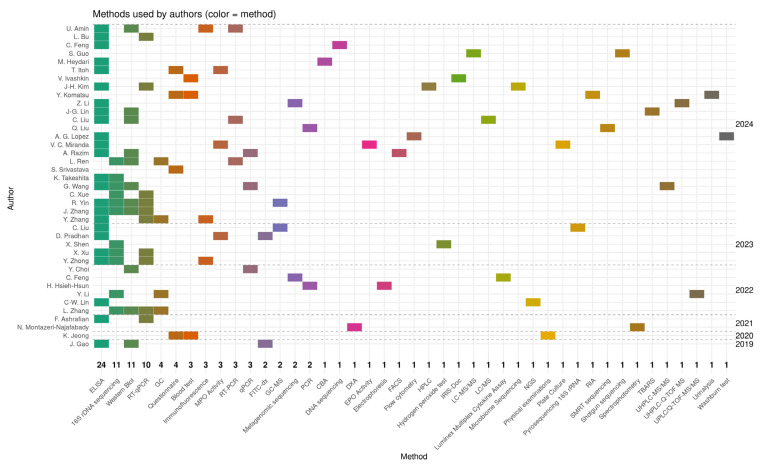
The heat map illustrates the research methodologies used by the authors in the publications analysed between 2019 and 2024. The values on the X axis indicate the number of publications in which the method was used [24,25,26,27,28,29,30,31,32,33,34,35,36,37,38,39,40,41,42,43,44,45,46,47,48,49,50,51,52,53,54,55,56,57,58,59,60,61,62].

Figure 4 illustrates the research methodologies employed by the authors in the publications analysed from 2019 to 2024. The figure presents a heat map where each rectangle’s colour corresponds to a specific analytical or molecular method, and the vertical axis lists the authors’ names organised by the year of publication. Among the techniques used, ELISA emerged as the most frequently employed method, appearing in 24 publications, underscoring its significance in quantifying cytokines and other immune biomarkers in studies on microbiota, probiotics, and postbiotics. Other prevalent methods included 16S rDNA sequencing, featured in 11 publications, which is extensively used to examine gut microbiota composition, and Western blotting, featured in 11 publications, primarily for detecting signalling proteins linked to inflammation or immune response. RT-qPCR and qPCR were used in four studies to assess gene expression and the presence of bacterial genetic material. The remaining methods appeared sporadically in only one or two publications and encompassed highly specialised techniques, such as LC-MS/MS, UPLC-Q-TOF-MS, metagenomic sequencing, SMRT sequencing, microbiome assays, and spectrophotometry. This diversity of methods reflects a broad spectrum of research approaches, ranging from classical immunological analyses to advanced omics techniques, including metabolomics, metagenomics, and transcriptomics. A notable increase in unique techniques was observed in 2023–2024 publications, indicating escalating research complexity and advancing methodological specialisation. Integrated analytical approaches that combine molecular methods with metabolite profiling and next-generation sequencing are becoming increasingly prevalent. The analysis of research methodologies revealed a predominance of techniques related to immunology and microbiota analysis, such as ELISA and 16S rDNA, while also expanding the methodological spectrum toward advanced molecular and omics analyses. This trend suggests a growing need for multilevel analyses of the intricate interactions between probiotics, microbiota, and host responses.

### 3.2. Risk of Bias

**Figure 5:**
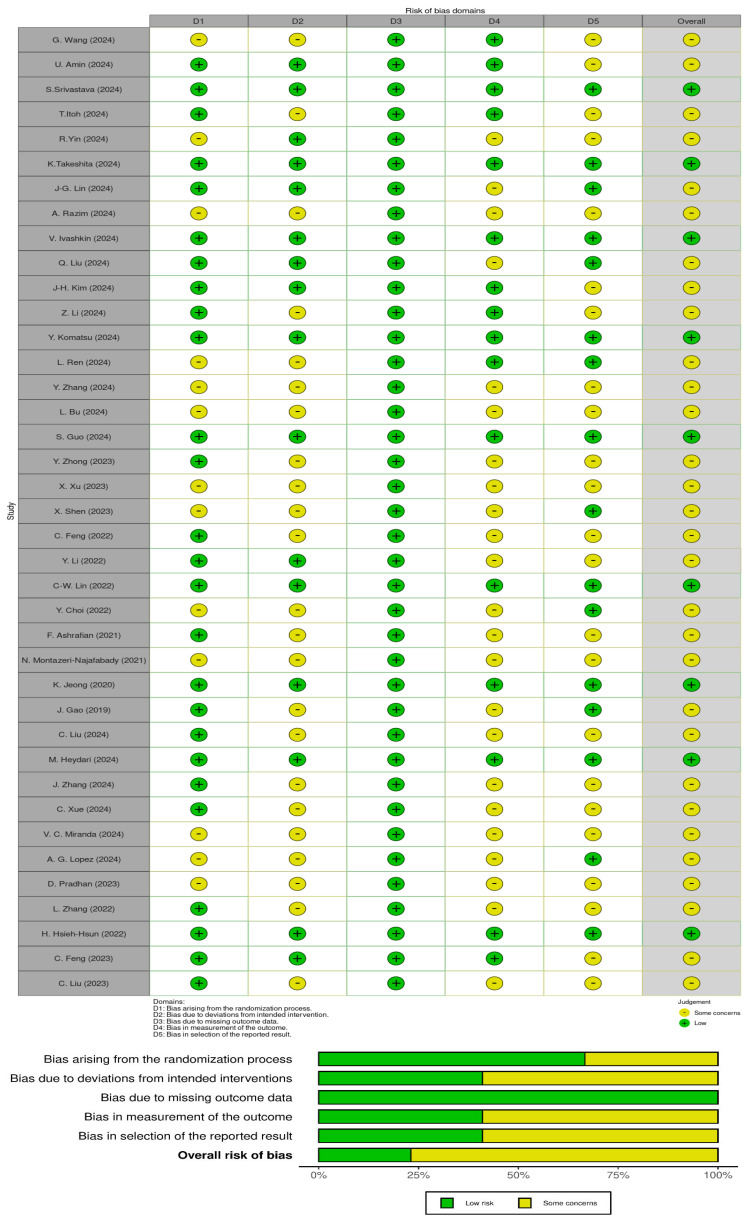
Risk of bias in the included studies [24,25,26,27,28,29,30,31,32,33,34,35,36,37,38,39,40,41,42,43,44,45,46,47,48,49,50,51,52,53,54,55,56,57,58,59,60,61,62].

The risk of bias in the included studies, shown in Figure 5, indicates that the overall risk of bias was a concern. Not all studies described the generation of random sequences in detail, and 26 reported correct allocation concealment methods. Most studies had an overall risk of bias owing to deviations from the intended intervention. All studies had a low risk of bias regarding missing outcome data. In contrast, only 16 studies had a low risk of bias for outcome measures. For most studies, the risk of reporting bias was unclear.

## 4. Discussion

The findings of this systematic review offer a comprehensive examination of the current understanding of the formulation of postbiotics and their application in the treatment and prevention of inflammation. Postbiotics are considered safer than probiotics because they do not require the presence of live microorganisms to exert beneficial effects on health. Therefore, the risk of interaction between inactivated microorganisms and host microbiota is eliminated [63]. Studies have highlighted the increasing interest in the therapeutic potential of postbiotics as a safe and stable alternative to probiotics, especially in vulnerable populations, such as individuals with compromised intestinal barriers or immunosuppression [1,16].

Research indicates that postbiotics can effectively modulate the immune response, demonstrating anti-inflammatory, immunomodulatory, and protective effects on intestinal epithelial integrity [11,13]. The composition of postbiotic preparations is contingent on the bacterial strain, method of inactivation, and processing technique employed. The most frequently utilised bacteria belong to the genera *Lactobacillus* (e.g., *L*. *plantarum* and *L*. *casei*) and *Bifidobacterium* (e.g., *B. animalis*), whereas Gram-negative bacteria, such as *Akkermansia muciniphila*, are employed less frequently [64,65]. Strain selection determines the composition of the bioactive components. Preparations derived from Gram-positive bacteria, such as *Lactobacillus* and *Bifidobacterium*, are rich in metabolites such as SCFAs, peptides, and EPS, which contribute to their anti-inflammatory and immunomodulatory properties. Conversely, postbiotics derived from Gram-negative bacteria, although used less frequently, contain bioactive membrane components (e.g., LPS) that exhibit immunoregulatory activity [17].

The presence of a variety of active substances in the cellular composition of postbiotics makes the selection of an appropriate microbial inactivation method, tailored to the specific strain, a key aspect to maintain the integrity and biological activity of these components. The predominance of heat inactivation as the primary processing technique, utilised in 84.6% of the cases analysed, indicates its practicality and scalability in postbiotic production. However, alternative methods, including UV radiation, enzymatic lysis, and freeze-drying, warrant further exploration to determine their effects on the preservation of biological activity [66].

Research has elucidated that the mechanisms through which postbiotics exert their effects include the suppression of pro-inflammatory cytokine expression (IL-6 and TNF-α), activation of pattern recognition receptors (PRRs) such as TLR2 and TLR4, modification of signalling pathways including NF-κB, MAPK, and NLRP3, and enhancement of intestinal barrier function via the expression of tight junction proteins [4,67]. In certain contexts, postbiotics have been observed to influence the Treg/Th17 balance, which is of particular significance in the management of inflammatory bowel disease. This modulation of immune responses supports epithelial integrity and mitigates immune-mediated damage to the gut. Conversely, numerous studies have demonstrated that postbiotics exert anti-inflammatory effects by inhibiting signalling pathways [68]. Postbiotics, such as sodium butyrate, have shown promise in glycaemic control by improving islet morphology and downregulating the NF-κB–mediated inflammatory signalling pathway in streptozotocin-induced T1D mice. This indicates that postbiotics can attenuate inflammation by suppressing key pro-inflammatory signalling [69]. Postbiotics have been shown to significantly modulate the composition of gut microbiota. Postbiotic interventions have resulted in an enriched composition of beneficial gut bacteria, including *B. animalis*, *L. salivarius*, and *A. muciniphila*. These bacteria contribute to gut barrier integrity and immune homeostasis, indirectly supporting intestinal epithelial protection [16,64]. Closely related to this effect of postbiotics is protection against gut dysbiosis and leaky gut. By restoring microbiota balance and inhibiting pathogenic bacterial growth, postbiotics help prevent dysbiosis-induced damage to the intestinal mucosa, reducing endotoxin (LPS) translocation and systemic inflammation, which exacerbates T1D [69]. It has also been demonstrated that postbiotics containing microbial metabolites, such as SCFAs, can influence the balance of the Th1/Th2 immune response, which is crucial in the context of allergies and autoimmune diseases [63]. Other components of postbiotic preparations with proven beneficial effects include bacterial enzymes, tryptophan derivatives such as melatonin, fermentation products such as lactic acid and isocaproic acid, and secondary metabolites (e.g., colipterins from *E. coli* and thioredoxins from *S. boulardii*), which have demonstrated immunomodulatory properties that contribute to beneficial effects against IBD, both in vitro and in vivo [70]. Similarly, beneficial effects have been observed for EPS secreted by probiotic strains, such *as Lactobacillus helveticus* and *L. rhamnosus*, which ameliorate gut inflammation by enhancing antioxidant defences and supporting barrier integrity [71,72]. Finally, several low-molecular-weight components of postbiotic formulations also exhibit antioxidant properties by reducing reactive oxygen species (ROS) and enhancing the activity of antioxidant enzymes, including superoxide dismutase (SOD), catalase (CAT), and glutathione peroxidase (GPx). This activity mitigates oxidative stress, which exacerbates inflammation and epithelial damage [73]. Although most evidence supporting the therapeutic efficacy of postbiotics is derived from gastrointestinal studies, beneficial effects have also been observed in metabolic, dermatological, and neurological diseases [5].

The overall risk of bias found in the included studies raises doubts about the reliability of the results presented. Although all studies showed a low risk of bias related to missing outcome data, other domains showed significant methodological shortcomings. The process of random sequence generation was not consistently reported, and only 26 studies provided clear information on allocation concealment. The majority of studies showed some concern or high risk for deviations from the intended interventions, which may have affected the internal validity of the results. In addition, only 16 studies reported a low risk of bias in outcome measurement, and the risk of selective reporting remained unclear in many cases. These issues suggest that although some methodological aspects were adequately addressed, caution should be exercised when interpreting results.

This systematic review has several limitations. First, the significant heterogeneity among the included studies—particularly regarding postbiotic formulations, inactivation methods, types of inflammatory conditions, and outcome measures—limits the generalizability of the findings and precludes the possibility of conducting a meta-analysis. The diversity in study designs, intervention protocols, and analytical techniques (e.g., ELISA, 16S rRNA, qPCR, and omics approaches) poses challenges for directly comparing results, leading to variability and inconsistent conclusions. Additionally, methodological differences, such as the lack of standardisation in sample preparation, sequencing methods, and extraction techniques (e.g., centrifugation, ultrafiltration, chromatography, and mass spectrometry), further impede comparability across studies. A significant limitation is that the included studies involved many different inflammatory pathologies with different origins and mechanisms of action. Inflammation, which is a response of the immune system to a variety of harmful agents, such as pathogens, damaged cells, or toxins, can take different forms. This pathophysiological heterogeneity may cause variability in the response to postbiotics and introduce potential bias into the results of the review. The different mechanisms and severity of inflammation in individual conditions make it difficult to directly compare therapeutic effects and to interpret the data at an overall level.

Second, most of the included studies were preclinical or animal-based, which restricts the applicability of findings to human health outcomes. The limited number of clinical trials, along with small sample sizes and short intervention periods, weakens the strength of the evidence and limits the ability to draw robust, evidence-based recommendations.

Third, many studies did not clearly report randomisation procedures or allocation concealment, increasing the risk of bias. Despite the use of rigorous selection criteria, these reporting deficiencies raise concerns about internal validity.

Finally, publication bias may be present, as studies with positive outcomes are more likely to be published, potentially leading to an overestimation of the therapeutic potential of postbiotics. Therefore, well-designed, large-scale randomised controlled trials with long-term follow-up and standardised methodologies are urgently needed to validate the efficacy and safety of postbiotic interventions in chronic inflammatory conditions.

Based on the data obtained, postbiotics should be regarded as promising adjunctive therapy components and potential functional food components. Their stability, safety, and capacity for precise formulation render them particularly valuable nutraceutical products. Future efforts should focus on developing standards for determining the content of active components, standardising inactivation methods, and conducting long-term, multicentre clinical trials. Additionally, it is recommended to analyse the synergistic effects of different strains and integrate clinical studies with metagenomic and metabolomic data to enhance the understanding of the interactions between postbiotics, microbiota, and the host immune system. It is particularly important to consider the individual characteristics of the microbiome and the inflammatory state of the patient to facilitate the creation of personalised postbiotic therapies. The implementation of such approaches may contribute not only to improving the health of patients with chronic inflammatory conditions but also to the development of effective preventive strategies for health and nutrition policies.

In contemporary nutrition and dietetics, postbiotics are emerging as pivotal components of the next generation of functional ingredients. Their primary advantage over probiotics is the elimination of risks associated with live microorganisms, which is particularly significant for individuals with dysbiosis, autoimmune diseases, or compromised immune systems [1]. Postbiotics are increasingly being considered for integration into standard dietary interventions and as adjuncts in the pharmacotherapy of inflammatory and metabolic diseases. Owing to their chemical stability and resilience to varying storage conditions, postbiotics can be seamlessly incorporated into diverse food matrices, ranging from fermented dairy products to capsules and powder formulations. This characteristic also enhances their commercial appeal compared to probiotics, which necessitate the viability of microorganisms until consumption. From a biotechnological standpoint, postbiotics present novel opportunities for optimising fermentation processes and designing bioactive ingredients through metabolic engineering. Notable examples include formulations aimed at producing specific metabolites, such as butyrate, propionate, and bioactive peptides, which possess anti-inflammatory, antitumour, and neuroprotective properties [13,17].

However, despite their great application potential, the implementation of postbiotics in clinical and dietary practices poses several normative and technological challenges. Currently, there is a lack of clear regulatory guidelines specifying the safety, efficacy, and labelling requirements for postbiotic products. Therefore, interdisciplinary cooperation among microbiologists, biotechnologists, nutritionists, and regulators is required to create standards to ensure the quality and efficacy of formulations available on the market. In the near future, it will be important to develop translational research that combines molecular analyses with clinical observations to better understand the relationship between postbiotic ingredients, microbiota, and host response.

## 5. Conclusions

This systematic review provides compelling evidence supporting the potential of postbiotics as safe and effective agents for the prevention and treatment of inflammation-related diseases. The absence of live microorganisms in postbiotic formulations eliminates the risk of adverse host–microbiota interactions, making them particularly suitable for vulnerable populations, such as immunocompromised individuals. This review aimed to identify patterns in formulation strategies, classify reported outcomes, and assess the quality of evidence. The most frequently used microorganisms belong to the *Lactobacillaceae* and *Bifidobacteriaceae* families. The results of this review indicate that the formulation of postbiotic preparations plays a critical role in determining their therapeutic potential for the management of chronic inflammation. Key factors influencing the efficacy of postbiotics include the selection of microbial strains, methods of inactivation, and processing conditions applied.

Based on the reviewed studies, thermal inactivation emerged as the most commonly employed technique, likely because of its operational simplicity and scalability. Nonetheless, given the susceptibility of cellular components to thermal degradation and the importance of preserving their biological activity, alternative inactivation methods (although less frequently utilized) should also be considered, as they were reported in several of the analysed publications.

Therapeutically, postbiotics exert multifaceted anti-inflammatory effects by modulating cytokine expression, influencing immune cell signalling pathways, and reinforcing epithelial barrier integrity. Their ability to regulate key immune mechanisms, such as Th1/Th2 and Treg/Th17 balance, makes them promising candidates for adjunctive treatment of chronic inflammatory conditions, including inflammatory bowel disease, metabolic syndrome, and autoimmune diseases. These findings underscore the importance of strain-specific and process-tailored formulation strategies to maximise the therapeutic efficacy of postbiotics in clinical settings.

However, many studies have methodological limitations, and the overall risk of bias is concerning. Future research should focus on standardising postbiotic preparations, conducting long-term clinical trials, and analysing the synergistic effects of different strains.

## Figures and Tables

**Table 1:** Characteristics of studies included in the systematic review.

*N*	Author	Study Design	Population Sample Size	Disease	Species/Strain	Inactivation	Formulation	Dosage Period	Administrations	Mechanism
**Animal Studies**										
1	Wang et al., 2024 [24]	Experimental study	Mice 5 groups *n* = 25, x ≥ 4 per groups	Dextran sodium sulfate-induced [DDS] colitis	*L*. *reuteri* I5007	Heat-killed (95 °C, 20 min)	Fluid	Daily for 1 week	Orally	modulation of the intestinal microbiota effect on tryptophan metabolism leading to production of ligands for the aryl hydrocarbon receptor
2	Amin et al., 2024 [25]	Experimental study	Mice 5 groups x ≥ 5 per groups	Osteoarthritis	*S*. *thermophilus* *L*. *pentosus*	Freeze-thaved (−80 °C)	Suspension	3 times a week for 6 weeks	Orally gavage	modulation of the immune response decreased expression of pro-inflammatory cytokines (IL-6) increased expression of anti-inflammatory markers (IL-4 and IL-10)
3	Itoh et al., 2024 [26]	Experimental study	Mice 8 groups *n* = 48, 6 per groups	Dextran sodium sulfate-induced [DDS] colitis	*L*. *argentoratensis BBLB001*	Heat-killed (no parameters)	Capsules	For 19 days	Orally	reduced inflammatory cytokine levels in the serum and colon tissues, colon shortening, and myeloperoxidase activation increased the gene and protein expressions of cell adhesion molecules in the colon tissue increased mucin expression and secretory IgA concentration in colon tissues
4	Yin et al., 2024 [27]	Experimental study	Mice 5 groups *n* = 36, 3–7 per groups	Alcohol liver disease	*L*. *johnsonii*	Heat-killed (85 °C, 15 min)	Suspension	Daily for 2 weeks	Intragastric gavage	activation of the NOD2-IL-22 signalling pathway in intestinal immune cells regulation of the STAT3 pathway in the liver helps correct the gut microbiota dysbiosis, specifically by reversing the reduction of butyrate-producing bacteria
5	Lin et al., 2024 [28]	Experimental study	Mice 6 gropus *n* = 36, 6 per groups	Acute kidney injury	*P*. *acidilactici* GKA4	Heat-killed (121 °C, 15 min)	Suspension	Daily for 10 days	Orally	antioxidant effect modulation of the immune response improvement of renal function by reducing levels of markers of kidney damage
6	Razim et al., 2024 [29]	Experimental study	Mice 4 groups *n* = 20, 5 per groups	Nosocomial infections diarrhea	*E*. *coli* O83	Ultracentrifugation (150,000× *g*/3 h/temp. 4 °C)	Suspension	No data	Intranasally	increased expression of genes associated with the TLR4 signalling pathway modulation of lung cell composition production of pro- and anti-inflammatory cytokines in cells
7	Kim et al., 2024 [30]	Experimental study	Mice 4 groups *n* = 20, 5 per groups	Type 2 diabetes	*L*. *plantarum* LRCC5314	Heat-killed (no parameters)	Suspension	For 12 weeks	Orally	influence on the modulation of the intestinal microbiota reduction of stress hormone levels improvement of the immune response
8	Li et al., 2024 [31]	Experimental study	Rats 4 groups *n* = 48, 12 per groups	Dextran sodium sulfate-induced [DDS] colitis	*B*. *longum* subsp. *infantis* B8762	Heat-killed (121 °C, 15 min)	Suspension	For 14 days	Orally	regulation of the production of cytokines and other inflammatory mediators support of the intestinal barrier integrity influence on the composition and diversity of the intestinal microbiota influence the production of metabolites in the intestines
9	Ren et al., 2024 [32]	Experimental study	Mice 5 groups *n* = 50, 10 per groups	Hyperuricaemia	*P*. *acidilactici* GQ01	Heat-killed (65 °C, 30 min)	Suspension	For 21 days	Orally gavage	inhibits the activity of the enzyme xanthine oxidase (XOD) restores balance to the intestinal microbiota increases the level of short-chain fatty acids influences the expression of genes and proteins related to renal reabsorption and excretion of uric acid
10	Zhang et al., 2024 [33]	Experimental study	Mice 2 groups *n* = 90	Alzheimer’s disease	*S*. *thermophilus* MN-ZLW-002	Heat-killed (65 °C, 2 h)	Suspension	Daily for 12 weeks	Orally gavage	modulation of antioxidant enzyme levels, BDNF mRNA and inflammatory markers in the hippocampus
11	Bu et al., 2024 [34]	Experimental study	Mice 5 groups *n* = 50, 10 per groups	Dextran sodium sulfate-induced [DDS] colitis	*L*. *rhamnosus* 2016SWU.05.0601	Heat-killed (100 °C, 30 min)	Suspension	For 5 weeks	Orally	modulation of the intestinal flora regulation of short-chain fatty acid levels immunomodulatory effect antioxidant properties
12	Zhong et al., 2023 [35]	Experimental study	Mice 4 groups *n* = 40, 10 per groups	Dextran sodium sulfate-induced [DDS] colitis	*C*. *crustorum* MN047 (CC)	Heat-killed (95 °C, 30 min)	Suspension	For 6 days	Orally gavage	alleviating the pathologic lesions of UC ameliorating the colonic inflammation attenuating the oxidative damage mitigating the damage of gut barrier modulating gut microbiota structure
13	Xu et al., 2023 [36]	Experimental study	Mice 5 groups *n* = 50, 10 per groups	Dextran sodium sulfate-induced [DDS] colitis	*S*. *boulardii*	Heat-killed (121 °C, 15 min)	Suspension	Daily for 7 days	Orally gavage	increased the expression of intestinal tight junction protein reduced the secretion of pro-inflammatory factors increased the secretion of anti-inflammatory factors maintained the homeostasis of intestinal microorganisms
14	Feng et al., 2022 [37]	Experimental study	Mice 3 groups *n* = 24, 8 per groups	Dextran sodium sulfate-induced [DDS] colitis	*B*. *bifidum* B1628	Heat-killed (95 °C, 15 min)	Suspension	Daily for 10 days	Orally	modulation of the balance of pro- and anti-inflammatory cytokines reduction of tissue damage in the large intestine regulation of the intestinal microbiota
15	Li et al., 2022 [38]	Experimental study	Mice 3 groups *n* = 88	Hypercholesterolemic	*L*. *plantarum* H6	Heat-killed (90 °C, 30 min)	Suspension	Daily for 4 weeks	Orally	influences the composition of the intestinal microbiota regulation of metabolites related to lipid metabolism ability to lower levels of total cholesterol, triglycerides, and low-density lipoprotein
16	Choi et al., 2022 [39]	Experimental study	Rats 4 groups *n* = 32, 8 per groups	Periodontitis	*L*. *fermentum* SMFM2017-CK1 *L*. *plantarum* SMFM2017-NK2 *P*. *pentosaceus* SMFM2017-NK1 *L*. *plantarum* SMFM2017-NK1 *L*. *paraplantarum* SMFM2017-YK1 *L*. *plantarum* SMFM2017-YK1 *L*. *fermentum* SMFM2017-NK1	Heat-killed (80 °C, 1 min)	Suspension	Daily for 8 weeks	Orally	antimicrobial activity against pathogens decreased levels of pro-inflammatory cytokines (TNF-α, IL-6) and increased levels of anti-inflammatory cytokines (IL-10) increased expression of genes encoding antioxidant enzymes anti-inflammatory effects
17	Ashrafian et al., 2021 [40]	Experimental study	Mice 5 groups *n* = 35, 7 per groups	Obesity	*A*. *muciniphila* MucT	Heat-killed (70 °C, 30 min)	Suspension	Daily for 5 weeks	Orally	impact on improving metabolic health by modulating the composition of the gut microbiota reducing inflammation
18	Montazeri-Najafabad et al., 2021 [41]	Experimental study	Rats *n* = 84	Post-menopausal osteoporosis	*L*. *acidophilus* *L*. *reuteri* *L*. *casei* *B*. *longum* *B*. *coagulans*	Heat-killed (60 °C, 30 min)	Suspension	Daily for 4 weeks	Orally	effect on improving bone metabolism improvement of bone mineral density
19	Gao et al., 2019 [42]	Experimental study	Mice 3 groups *n* = 24, 8 per groups Rats 2 groups *n* = 12, 6 per groups	Dextran sodium sulfate-induced [DDS] colitis Acute liver failure	*L*. *rhamnosus* GG (LGG)	Killed by enzyme (Proteinase K)	Pectin-zein beads	Daily for 3 days	Orally	improving the integrity of the intestinal barrier stimulation of the immune response
20	Liu et al., 2024 [43]	Experimental study	Mice 7 groups *n* = 42, 6 per groups	Alcohol liver disease	*L*. *reuteri*	Heat-killed (65 °C, 30 min)	Suspension	Daily for 23 days	Orally gavage	modulation of FXR/SHP/SREBP-1c signaling pathway improvement of lipid metabolism reduction of hepatic steatosis
21	Zhang et al., 2024 [44]	Experimental study	Mice 5 groups *n* = 60, 12 per groups	Dextran sodium sulfate-induced [DDS] colitis	*L*. *rhamnosus* 1.0320	Heat-killed (121 °C/15 min)	Suspension	Daily for 14 days	Orally gavage	signs of reductions in the disease activity index amelioration of colon tissue damage decreased secretion of pro-inflammatory cytokines reduced oxidative stress levels lower bone marrow peroxidase activity
22	Xue et al., 2024 [45]	Experimental study	Mice 6 groups *n* = 30, 5 per groups	Constipation	*L*. *paracasei*	Heat-killed (no parameters)	Fluid	2 times for 14 days	Orally	alleviated colonic inflammation regulate the intestinal flora downregulate the abundance of *Proteobacteria* upregulate the abundance of species of *Firmicutes* and *Actinobacteriota*
23	Miranda et al., 2024 [46]	Experimental study	Mice 5 groups *n* = 30, 6 per groups	Food allergy	*A*. *muciniphila* BAA-835	Heat-killed (70 °C/10 min)	Suspension	Daily for 14 days	Intragastric gavage	ability to reduce levels of pro-inflammatory cytokines reduce *Staphylococcus* levels and yeast incidence in the gut microbiome reduction in anti-OVA IgE antibody levels and eosinophil recruitment
24	Lopez et al., 2024 [47]	Experimental study	Mice 3 groups *n* = 18, 6 per groups	Emergency myelopoiesis	*L*. *rhamnosus* CRL1505	Ultraviolet radiation (2 h)	Suspension	Daily for 5 days	Orally	increase blood neutrophils and peroxidase-positive cells, while improving cytokine production and phagocytic activity of alveolar macrophages
25	Pradhan et al., 2023 [48]	Experimental study	Mice 5 groups *n* = 20, 10 per groups	Dextran sodium sulfate-induced [DDS] colitis	*L*. *plantarum* MTCC 5690 *L*. *fermentum* MTCC 5689 *L*. *rhamnosus* GG (LGG)	Microfluidizer lysis (15 min)	Fluid	For 14 days	Orally	decreases the secretion of TNF-α increases IL-10 secretion
26	Zhang et al., 2022 [49]	Experimental study	Mice 4 groups *n* = 32, 8 per groups	Hyperuricemia	*A*. *muciniphila*	Heat-killed (70 °C/30 min)	Suspension	For 3 weeks	Orally	influence on uric acid metabolism inhibits xanthine oxidase activity modulates inflammation and composition of intestinal microbiota
27	Feng et al., 2023 [50]	Experimental study	Rat 5 groups *n* = 40, 8 per groups	Dextran sodium sulfate-induced [DDS] colitis	*L*. *casei* Zhang, *L*. *plantarum* P-8 *B*. *animalis* subsp. *lactis* V9	Heat-killed (95 °C/60 min)	Suspension	For 14 days	Orally gavage	improvement of intestinal homeostasis reduction of intestinal tissue damage influence on the metabolism of the intestinal microbiota
28	Liu et al., 2024 [51]	Experimental study	Mice 5 groups *n* = 40, 8 per groups	Dextran sodium sulfate-induced [DDS] colitis	*L*. *fermentum* HF06	Heat-killed (121 °C/15 min)	Suspension	Daily for 24 days	Orally gavage	reduces pro-inflammatory cytokines (IL-6, TNF-α, and IL-1β) increases anti-inflammatory cytokine IL-10 modulation of gut microbiota enhancement of short-chain fatty acid production increases antioxidant enzyme levels
**Human Studies**										
29	Ivashkin et al., 2024 [52]	Randomized, double-blind, placebo-controlled study	Human 2 groups *n* = 129	*Helicobacter pylori* in adults with functional dyspepsia	*L*. *reuteri* DSM17648	Spray-dried (no parameters)	Capsules	2 times for 14 days	Orally	reduce the mobility and ability to adhere to the gastric mucosa of *Helicobacter pylori* reduce side effects associated with *H*. *pylori* eradication therapy
30	Liu et al., 2023 [53]	Randomized controlled study	Human 3 groups *n* = 98	Dental caries	*L*. *casei* Zhang *L*. *plantarum* P-8 *B*. *animalis* subsp. *lactis* V9	Heat-killed (75 °C, 15 min)	Pill	2 times a day for 14 days	Orally	influences changes in metabolic pathways (association with RuMP cycle and geranylgeranyl diphosphate biosynthesis)
31	Komatsu et al., 2024 [54]	Randomized, placebo-controlled, double-blind, parallel-group clinical study	Human 2 groups *n* = 120, 60 per groups	Gastroesophageal reflux disease functional dyspepsia	*L*. *johnsonii* No. 1088	Heat-killed (no parameters)	Sachet to drink	Daily for 6 weeks	Orally	modulation of the intestinal microbiota effects on gut–brain interactions
32	Guo et al., 2024 [55]	Randomized, double-blind, placebo-controlled crossover study	Human *n* = 69	Chronic diarrhea	*L*. *casei* Zhang *L*. *plantarum* P-8 *B*. *animalis* subsp. *lactis* V9	Heat-killed (95 °C, 60 min)	Pill	Daily for 21 days	Orally	production of short-chain fatty acids modulation of the intestinal microbiota influence on tryptophan metabolism
33	Shen et al., 2023 [56]	Clinical study	Human *n* = 50	Bacterial vaginitis	*L*. *paracasei* *L*. *rhamnosus*	Heat-killed (no parameters)	Gel	Daily for 7 days	Vaginally	improving the vaginal microbiota increasing *Lactobacilli* levels reduction of potential pathogens
34	Lin et al., 2022 [57]	Experimental study	Human 3 groups *n* = 75, 25 per groups	Dental caries	*L*. *salivarius* subsp. *salicinius* AP 32 *L*. *paracasei* ET-66 *L* *L*. *plantarum* LPL28	Heat-killed (135 –140 °C, 4 s)	Lozenge	Daily for 4 weeks	Orally	ability to reduce colonization and growth of oral pathogens and bacteria associated with periodontal disease effect on the growth of beneficial microorganisms increases the concentration of IgA in saliva production of short-chain fatty acids
35	Jeong et al., 2020 [58]	Randomized, double-blind, placebo-controlled parallel study	Human *n* = 120	Atopic dermatitis	*L*. *rhamnosus* IDCC 3201	Heat-killed (no parameters)	Suspension	Daily for 12 weeks	Orally	modulation of the immune response decreases in levels of eosinophil cationic protein and interleukin-31 support of the gut microbiota and skin barrier integrity
36	Heydari et al., 2024 [59]	Randomized, placebo-controlled, triple-masking clinical trial	Human 4 groups *n* = 40, 10 per groups	Dry eye disease	*L*. *sakei*	Heat-killed (121 °C)	Suspension	Daily for 4 weeks	Eye drops	alleviate the signs and symptoms of DED suppress the inflammatory response on the ocular surface reducing levels of inflammatory cytokines (IL-6, TNF-α, IFN-γ)
37	Ho et al., 2022 [60]	Randomized clinical study	Human *n* = 20	Acne vulgaris	*L*. *salivarius* AP-32 *L*. *acidophilus* TYCA06 *L*. *reuteri* GL-104 *B*. *animalis* subsp. *lactis CP-9* *B*. *longum subsp. infantis* BLI-02, *B*. *longum* subsp. *infantis* OLP-01 *B*. *breve* Bv-889, *B*. *bifidum* VDD088 *B*. *bifidum* Bf-688 *S*. *thermophilus SY-66*	Heat-killed (95 °C/20 min)	Gel	For 4 weeks	Facial skin	ability to inhibit the growth of skin pathogens reduction of inflammation in skin diseases improvement of skin regeneration support of the microbiota on the skin influence on the body’s immune response
38	Srivastava et al., 2024 [61]	Randomized double-blind, placebo-controlled study	Human *n* = 200	Diarrhea-predominant irritable bowel syndrome	*B*. *longum* CECT 7347 ES1	Heat-killed (no parameters)	Capsules	Daily for 12 weeks	Orally	reducing the severity of IBS symptoms improvement of intestinal microbiota balance regulation of the immune response
39	Takeshita et al., 2024 [62]	Pilot, double-blind, randomized, placebo-controlled study	Human *n* = 41	Respiratory tract infections in premature infants	*P*. *acidilactici* K15	Heat-killed (90 °C)	Suspension	Daily for 1 year	Orally	increase in the number of bacteria of the genus *Faecalimonas* increasing the production of immunoglobulin A (IgA) by B cells activation of innate immunity influencing T- and B-cell functions through the secretion of cytokines and costimulatory molecules

## References

[1] Salminen S., Collado M.C., Endo A., Hill C., Lebeer S., Quigley E.M.M., Sanders M.E., Shamir R., Swann J.R., Szajewska H. (2021). The International Scientific Association of Probiotics and Prebiotics (ISAPP) Consensus Statement on the Definition and Scope of Postbiotics. Nat. Rev. Gastroenterol. Hepatol..

[2] Aguilar-Toalá J.E., Garcia-Varela R., Garcia H.S., Mata-Haro V., González-Córdova A.F., Vallejo-Cordoba B., Hernández-Mendoza A. (2018). Postbiotics: An Evolving Term within the Functional Foods Field. Trends Food Sci. Technol..

[3] Żółkiewicz J., Marzec A., Ruszczyński M., Feleszko W. (2020). Postbiotics—A Step beyond Pre-and Probiotics. Nutrients.

[4] Taverniti V., Guglielmetti S. (2011). The Immunomodulatory Properties of Probiotic Microorganisms beyond Their Viability (Ghost Probiotics: Proposal of Paraprobiotic Concept). Genes Nutr..

[5] Cicenia A., Scirocco A., Carabotti M., Pallotta L., Marignani M., Severi C. (2014). Postbiotic Activities of Lactobacilli-Derived Factors. J. Clin. Gastroenterol..

[6] Tsilingiri K., Rescigno M. (2013). Postbiotics: What Else?. Benef. Microbes.

[7] Bourebaba Y., Marycz K., Mularczyk M., Bourebaba L. (2022). Postbiotics as Potential New Therapeutic Agents for Metabolic Disorders Management. Biomed. Pharmacother..

[8] da Silva Vale A., de Melo Pereira G.V., de Oliveira A.C., de Carvalho Neto D.P., Herrmann L.W., Karp S.G., Soccol V.T., Soccol C.R. (2023). Production, Formulation, and Application of Postbiotics in the Treatment of Skin Conditions. Fermentation.

[9] Abbasi A., Rad A.H., Ghasempour Z., Sabahi S., Kafil H.S., Hasannezhad P., Rahbar Saadat Y., Shahbazi N. (2022). The Biological Activities of Postbiotics in Gastrointestinal Disorders. Crit. Rev. Food Sci. Nutr..

[10] Wang Y., Kasper L.H. (2014). The Role of Microbiome in Central Nervous System Disorders. Brain Behav. Immun..

[11] Kavita, Om H., Chand U., Kushawaha P.K. (2024). Postbiotics: An Alternative and Innovative Intervention for the Therapy of Inflammatory Bowel Disease. Microbiol. Res..

[12] Chen L., Deng H., Cui H., Fang J., Zuo Z., Deng J., Li Y., Wang X., Zhao L. (2017). Inflammatory Responses and Inflammation-Associated Diseases in Organs. Oncotarget.

[13] Zhao X., Liu S., Li S., Jiang W., Wang J., Xiao J., Chen T., Ma J., Khan M.Z., Wang W. (2024). Unlocking the Power of Postbiotics: A Revolutionary Approach to Nutrition for Humans and Animals. Cell Metab..

[14] Tsilingiri K., Barbosa T., Penna G., Caprioli F., Sonzogni A., Viale G., Rescigno M. (2012). Probiotic and Postbiotic Activity in Health and Disease: Comparison on a Novel Polarised Ex-Vivo Organ Culture Model. Gut.

[15] Cuevas-González P.F., Liceaga A.M., Aguilar-Toalá J.E. (2020). Postbiotics and Paraprobiotics: From Concepts to Applications. Food Res. Int..

[16] Liang B., Xing D. (2023). The Current and Future Perspectives of Postbiotics. Probiotics Antimicrob. Proteins.

[17] Ratajczak W., Rył A., Mizerski A., Walczakiewicz K., Sipak O., Laszczyńska M. (2019). Immunomodulatory Potential of Gut Microbiome-Derived Shortchain Fatty Acids (SCFAs). Acta Biochim. Pol..

[18] Wang S., Wang P., Wang D., Shen S., Wang S., Li Y., Chen H. (2024). Postbiotics in Inflammatory Bowel Disease: Efficacy, Mechanism, and Therapeutic Implications. J. Sci. Food Agric..

[19] Page M.J., Moher D., Bossuyt P.M., Boutron I., Hoffmann T.C., Mulrow C.D., Shamseer L., Tetzlaff J.M., Akl E.A., Brennan S.E. (2021). PRISMA 2020 Explanation and Elaboration: Updated Guidance and Exemplars for Reporting Systematic Reviews. BMJ.

[20] Jan van Eck N., Waltman L. VOSviewer Manual. https://www.vosviewer.com/documentation/Manual_VOSviewer_1.6.13.pdf.

[21] Ouzzani M., Hammady H., Fedorowicz Z., Elmagarmid A. (2016). Rayyan-a Web and Mobile App for Systematic Reviews. Syst. Rev..

[22] Sterne J.A.C., Savović J., Page M.J., Elbers R.G., Blencowe N.S., Boutron I., Cates C.J., Cheng H.Y., Corbett M.S., Eldridge S.M. (2019). RoB 2: A Revised Tool for Assessing Risk of Bias in Randomised Trials. BMJ.

[23] McGuinness L.A., Higgins J.P.T. (2021). Risk-of-Bias VISualization (Robvis): An R Package and Shiny Web App for Visualizing Risk-of-Bias Assessments. Res. Synth. Methods.

[24] Wang G., Fan Y., Zhang G., Cai S., Ma Y., Yang L., Wang Y., Yu H., Qiao S., Zeng X. (2024). Microbiota-Derived Indoles Alleviate Intestinal Inflammation and Modulate Microbiome by Microbial Cross-Feeding. Microbiome.

[25] Amin U., Jiang R., Raza S.M., Fan M., Liang L., Feng N., Li X., Yang Y., Guo F. (2024). Gut-Joint Axis: Oral Probiotic Ameliorates Osteoarthritis. J. Tradit. Complement. Med..

[26] Srivastava S., Basak U., Naghibi M., Vijayakumar V., Parihar R., Patel J., Jadon P.S., Pandit A., Dargad R.R., Khanna S. (2024). A Randomized Double-Blind, Placebo-Controlled Trial to Evaluate the Safety and Efficacy of Live Bifidobacterium Longum CECT 7347 (ES1) and Heat-Treated Bifidobacterium Longum CECT 7347 (HT-ES1) in Participants with Diarrhea-Predominant Irritable Bowel Syndrome. Gut Microbes.

[27] Itoh T., Miyazono D., Sugata H., Mori C., Takahata M. (2024). Anti-Inflammatory Effects of Heat-Killed Lactiplantibacillus Argentoratensis BBLB001 on a Gut Inflammation Co-Culture Cell Model and Dextran Sulfate Sodium-Induced Colitis Mouse Model. Int. Immunopharmacol..

[28] Yin R., Wang T., Sun J., Dai H., Zhang Y., Liu N., Liu H. (2024). Postbiotics from Lactobacillus Johnsonii Activates Gut Innate Immunity to Mitigate Alcohol-Associated Liver Disease. Adv. Sci..

[29] Takeshita K., Hishiki H., Takei H., Ikari N., Tanaka S., Iijima Y., Ogata H., Fujishiro K., Tominaga T., Konno Y. (2024). The Effectiveness of Heat-Killed Pediococcus Acidilactici K15 in Preventing Respiratory Tract Infections in Preterm Infants: A Pilot Double-Blind, Randomized, Placebo-Controlled Study. Nutrients.

[30] Lin J.G., Jiang W.P., Tsai Y.S., Lin S.W., Chen Y.L., Chen C.C., Huang G.J. (2024). Dietary Probiotic Pediococcus Acidilactici GKA4, Dead Probiotic GKA4, and Postbiotic GKA4 Improves Cisplatin-Induced AKI by Autophagy and Endoplasmic Reticulum Stress and Organic Ion Transporters. Nutrients.

[31] Razim A., Zabłocka A., Schmid A., Thaler M., Černý V., Weinmayer T., Whitehead B., Martens A., Skalska M., Morandi M. (2024). Bacterial Extracellular Vesicles as Intranasal Postbiotics: Detailed Characterization and Interaction with Airway Cells. J. Extracell. Vesicles.

[32] Ivashkin V., Maev I., Poluektova E., Sinitsa A., Avalueva E., Mnatsakanyan M., Simanenkov V., Karpeeva J., Kopylova D., Kuprina I. (2024). Efficacy and Safety of Postbiotic Contained Inactivated Lactobacillus Reuteri (Limosilactobacillus Reuteri) DSM 17648 as Adjuvant Therapy in the Eradication of Helicobacter Pylori in Adults with Functional Dyspepsia: A Randomized Double-Blind Placebo Controlled Trial. Clin. Transl. Gastroenterol..

[33] Liu Q., Ma T., Feng C., Li Y., Jin H., Shi X., Kwok L.-Y., Shi Y., Chen T., Zhang H. (2023). Adjuvant Postbiotic Administration Improves Dental Caries Prognosis by Restoring the Oral Microbiota. Food Sci. Human Wellness.

[34] Kim J.H., Kwak W., Nam Y., Baek J., Lee Y., Yoon S., Kim W. (2024). Effect of Postbiotic Lactiplantibacillus Plantarum LRCC5314 Supplemented in Powdered Milk on Type 2 Diabetes in Mice. J. Dairy Sci..

[35] Li Z., Peng C., Sun Y., Zhang T., Feng C., Zhang W., Huang T., Yao G., Zhang H., He Q. (2024). Both Viable Bifidobacterium Longum Subsp. Infantis B8762 and Heat-Killed Cells Alleviate the Intestinal Inflammation of DSS-Induced IBD Rats. Microbiol. Spectr..

[36] Komatsu Y., Miura H., Iwama Y., Urita Y. (2024). Beneficial Effect of Heat-Killed Lactic Acid Bacterium Lactobacillus Johnsonii No. 1088 on Temporal Gastroesophageal Reflux-Related Symptoms in Healthy Volunteers: A Randomized, Placebo-Controlled, Double-Blind, Parallel-Group Study. Nutrients.

[37] Ren L., Wang S., Liu S., Prasanthi H.A.C., Li Y., Cao J., Zhong F., Guo L., Lu F., Luo X. (2024). Postbiotic of Pediococcus Acidilactici GQ01, a Novel Probiotic Strain Isolated from Natural Fermented Wolfberry, Attenuates Hyperuricaemia in Mice through Modulating Uric Acid Metabolism and Gut Microbiota. Foods.

[38] Zhang Y., Wang Y., Zhou Z., Yang Y., Zhao J., Kang X., Li Z., Shen X., He F., Cheng R. (2024). Live and Heat-Inactivated Streptococcus Thermophilus MN-ZLW-002 Mediate the Gut–Brain Axis, Alleviating Cognitive Dysfunction in APP/PS1 Mice. Nutrients.

[39] Bu L., Li Y., Wang C., Jiang Y., Suo H. (2024). Preventive Effect of Lacticaseibacillus Rhamnosus 2016SWU.05.0601 and Its Postbiotic Elements on Dextran Sodium Sulfate-Induced Colitis in Mice. Front. Microbiol..

[40] Guo S., Ma T., Kwok L.Y., Quan K., Li B., Wang H., Zhang H., Menghe B., Chen Y. (2024). Effects of Postbiotics on Chronic Diarrhea in Young Adults: A Randomized, Double-Blind, Placebo-Controlled Crossover Trial Assessing Clinical Symptoms, Gut Microbiota, and Metabolite Profiles. Gut Microbes.

[41] Zhong Y., Wang T., Wang X., Lü X. (2023). The Protective Effect of Heat-Inactivated Companilactobacillus Crustorum on Dextran Sulfate Sodium-Induced Ulcerative Colitis in Mice. Nutrients.

[42] Xu X., Wu J., Jin Y., Huang K., Zhang Y., Liang Z. (2023). Both Saccharomyces Boulardii and Its Postbiotics Alleviate Dextran Sulfate Sodium-Induced Colitis in Mice, Association with Modulating Inflammation and Intestinal Microbiota. Nutrients.

[43] Shen X., Xu L., Zhang Z., Yang Y., Li P., Ma T., Guo S., Kwok L.Y., Sun Z. (2023). Postbiotic Gel Relieves Clinical Symptoms of Bacterial Vaginitis by Regulating the Vaginal Microbiota. Front. Cell Infect. Microbiol..

[44] Feng C., Zhang W., Zhang T., He Q., Kwok L.Y., Tan Y., Zhang H. (2022). Heat-Killed Bifidobacterium Bifidum B1628 May Alleviate Dextran Sulfate Sodium-Induced Colitis in Mice, and the Anti-Inflammatory Effect Is Associated with Gut Microbiota Modulation. Nutrients.

[45] Li Y., Chen M., Ma Y., Yang Y., Cheng Y., Ma H., Ren D., Chen P. (2022). Regulation of Viable/Inactivated/Lysed Probiotic Lactobacillus Plantarum H6 on Intestinal Microbiota and Metabolites in Hypercholesterolemic Mice. npj Sci. Food.

[46] Lin C.W., Chen Y.T., Ho H.H., Kuo Y.W., Lin W.Y., Chen J.F., Lin J.H., Liu C.R., Lin C.H., Yeh Y.T. (2022). Impact of the Food Grade Heat-Killed Probiotic and Postbiotic Oral Lozenges in Oral Hygiene. Aging.

[47] Choi Y., Park E., Yoon Y., Ha J. (2022). Development of Postbiotics by Bioconverting Whey Using Lactobacillus Plantarum SMFM2017-YK1 and Limosilactobacillus Fermentum SMFM2017-NK1 to Alleviate Periodontitis. PLoS ONE.

[48] Ashrafian F., Keshavarz Azizi Raftar S., Lari A., Shahryari A., Abdollahiyan S., Moradi H.R., Masoumi M., Davari M., Khatami S., Omrani M.D. (2021). Extracellular Vesicles and Pasteurized Cells Derived from Akkermansia Muciniphila Protect against High-Fat Induced Obesity in Mice. Microb. Cell Fact..

[49] Montazeri-Najafabady N., Ghasemi Y., Dabbaghmanesh M.H., Ashoori Y., Talezadeh P., Koohpeyma F., Abootalebi S.N., Gholami A. (2021). Exploring the Bone Sparing Effects of Postbiotics in the Post-Menopausal Rat Model. BMC Complement. Med. Ther..

[50] Jeong K., Kim M., Jeon S.A., Kim Y.H., Lee S. (2020). A Randomized Trial of Lactobacillus Rhamnosus IDCC 3201 Tyndallizate (RHT3201) for Treating Atopic Dermatitis. Pediatr. Allergy Immunol..

[51] Gao J., Li Y., Wan Y., Hu T., Liu L., Yang S., Gong Z., Zeng Q., Wei Y., Yang W. (2019). A Novel Postbiotic from Lactobacillus Rhamnosus GG with a Beneficial Effect on Intestinal Barrier Function. Front. Microbiol..

[52] Liu C., Cai T., Cheng Y., Bai J., Li M., Gu B., Huang M., Fu W. (2024). Postbiotics Prepared Using Lactobacillus Reuteri Ameliorates Ethanol-Induced Liver Injury by Regulating the FXR/SHP/SREBP-1c Axis. Mol. Nutr. Food Res..

[53] Heydari M., Kalani M., Ghasemi Y., Nejabat M. (2024). The Effect of Ophthalmic and Systemic Formulations of Latilactobacillus Sakei on Clinical and Immunological Outcomes of Patients with Dry Eye Disease: A Factorial, Randomized, Placebo-Controlled, and Triple-Masking Clinical Trial. Probiotics Antimicrob. Proteins.

[54] Zhang J., Duan X., Chen X., Qian S., Ma J., Jiang Z., Hou J. (2024). Lactobacillus Rhamnosus 1.0320 Postbiotics Ameliorate Dextran Sodium Sulfate-Induced Colonic Inflammation and Oxidative Stress by Regulating the Intestinal Barrier and Gut Microbiota. J. Agric. Food Chem..

[55] Xue C., Li M., Luo M., Zhang B., Wang Y. (2024). Efficacy of Lacticaseibacillus Paracasei Fermented Milk on a Model of Constipation Induced by Loperamide Hydrochloride in BALB/c Mice. J. Food Sci..

[56] Miranda V.C., Souza R.O., Quintanilha M.F., Gallotti B., Assis H.C., Faria A.M.C., Nicoli J.R., Cara D.C., Martins F.S. (2024). A Next-Generation Bacteria (Akkermansia Muciniphila BAA-835) Presents Probiotic Potential Against Ovalbumin-Induced Food Allergy in Mice. Probiotics Antimicrob. Proteins.

[57] López A.G., Vasile B., Kolling Y., Ivir M., Gutiérrez F., Alvarez S., Salva S. (2024). Can Lacticaseibacillus Rhamnosus CRL1505 Postbiotic Improve Emergency Myelopoiesis in Immunocompromised Mice?. Microbes Infect..

[58] Pradhan D., Gulati G., Avadhani R., HM R., Soumya K., Kumari A., Gupta A., Dwivedi D., Kaushik J.K., Grover S. (2023). Postbiotic Lipoteichoic Acid of Probiotic Lactobacillus Origin Ameliorates Inflammation in HT-29 Cells and Colitis Mice. Int. J. Biol. Macromol..

[59] Zhang L., Liu J., Jin T., Qin N., Ren X., Xia X. (2022). Live and Pasteurized Akkermansia Muciniphila Attenuate Hyperuricemia in Mice through Modulating Uric Acid Metabolism, Inflammation, and Gut Microbiota. Food Funct..

[60] Ho H.H., Chen C.W., Yi T.H., Huang Y.F., Kuo Y.W., Lin J.H., Chen J.F., Tsai S.Y., Chan L.P., Liang C.H. (2022). Novel Application of a Co-Fermented Postbiotics of TYCA06/AP-32/CP-9/Collagen in the Improvement of Acne Vulgaris—A Randomized Clinical Study of Efficacy Evaluation. J. Cosmet. Dermatol..

[61] Feng C., Peng C., Zhang W., Zhang T., He Q., Kwok L.Y., Zhang H. (2023). Postbiotic Administration Ameliorates Colitis and Inflammation in Rats Possibly through Gut Microbiota Modulation. J. Agric. Food Chem..

[62] Liu C., Qi X., Li D., Zhao L., Li Q., Mao K., Shen G., Ma Y., Wang R. (2024). Limosilactobacillus Fermentum HF06-Derived Paraprobiotic and Postbiotic Alleviate Intestinal Barrier Damage and Gut Microbiota Disruption in Mice with Ulcerative Colitis. J. Sci. Food Agric..

[63] Mosca A., Abreu Y., Abreu A.T., Gwee K.A., Ianiro G., Tack J., Nguyen T.V.H., Hill C. (2022). The Clinical Evidence for Postbiotics as Microbial Therapeutics. Gut Microbes.

[64] Vera-Santander V.E., Hernández-Figueroa R.H., Jiménez-Munguía M.T., Mani-López E., López-Malo A. (2023). Health Benefits of Consuming Foods with Bacterial Probiotics, Postbiotics, and Their Metabolites: A Review. Molecules.

[65] Wegh C.A.M., Geerlings S.Y., Knol J., Roeselers G., Belzer C. (2019). Postbiotics and Their Potential Applications in Early Life Nutrition and Beyond. Int. J. Mol. Sci..

[66] Zhong Y., Wang T., Luo R., Liu J., Jin R., Peng X. (2024). Recent Advances and Potentiality of Postbiotics in the Food Industry: Composition, Inactivation Methods, Current Applications in Metabolic Syndrome, and Future Trends. Crit. Rev. Food Sci. Nutr..

[67] Jastrząb R., Graczyk D., Siedlecki P. (2021). Molecular and Cellular Mechanisms Influenced by Postbiotics. Int. J. Mol. Sci..

[68] Ying Z.H., Mao C.L., Xie W., Yu C.H. (2023). Postbiotics in Rheumatoid Arthritis: Emerging Mechanisms and Intervention Perspectives. Front. Microbiol..

[69] Zhang Y., Huang A., Li J., Munthali W., Cao S., Putri U.M.P., Yang L. (2024). The Effect of Microbiome-Modulating Agents (MMAs) on Type 1 Diabetes: A Systematic Review and Meta-Analysis of Randomized Controlled Trials. Nutrients.

[70] García Mansilla M.J., Rodríguez Sojo M.J., Lista A.R., Ayala Mosqueda C.V., Ruiz Malagón A.J., Ho Plagaro A., Gálvez J., Rodríguez Nogales A., Rodríguez Sánchez M.J. (2025). Microbial-Derived Antioxidants in Intestinal Inflammation: A Systematic Review of Their Therapeutic Potential. Antioxidants.

[71] Li B., Du P., Smith E.E., Wang S., Jiao Y., Guo L., Huo G., Liu F. (2019). In Vitro and in Vivo Evaluation of an Exopolysaccharide Produced by Lactobacillus Helveticus KLDS1.8701 for the Alleviative Effect on Oxidative Stress. Food Funct..

[72] Li J., Li Q., Gao N., Wang Z., Li F., Li J., Shan A. (2021). Exopolysaccharides Produced by: Lactobacillus Rhamnosus GG Alleviate Hydrogen Peroxide-Induced Intestinal Oxidative Damage and Apoptosis through the Keap1/Nrf2 and Bax/Bcl-2 Pathways in Vitro. Food Funct..

[73] Jalali S., Mojgani N., Sanjabi M.R., Saremnezhad S., Haghighat S. (2024). Functional Properties and Safety Traits of *L. Rhamnosus* and *L. Reuteri* Postbiotic Extracts. AMB Express.

